# Supplementary material for: Telacebec (Q203)-containing intermittent oral regimens sterilized mice infected with Mycobacterium ulcerans after only 16 doses
Source: PLoS Negl Trop Dis. 2020 Aug 31;14(8):e0007857. doi: 10.1371/journal.pntd.0007857 (PMC7494103; doi:10.1371/journal.pntd.0007857)
Supplement: Attachment — Submitted filename: Response to reviewers_PNTD D-19-01766.docx [file pntd.0007857.s001.docx]

**Responses to reviewer’s comments**

**Methods**

*Reviewer #1: These studies use a well-established mouse model of M. ulcerans infection which has been a useful guide to antibiotic treatment of human infection. The methods are well applied to this study of new antibiotic combinations by authors who are familiar with the mouse model. Well-designed study likely to yield clear answers. Ethical issues have been addressed.*

We thank the reviewer for this positive comment.

*Reviewer #2: 1) They did not show how they calculated "the M. ulcerans strain Cu001 (5.02 and 4.6 log10 in the 1st and 2nd experiment, respectively)." The right method should be calculating the CFUs from the mouse footpads on the day after infection.*

Answer: Indeed, this precision is lacking. We corrected the text as follows: “The number of living bacilli in the bacterial suspension used for inoculation has been determined to be 5.02 and 4.6 log_10_ in the 1^st^ and 2^nd^ experiment, respectively, by culturing the inoculum on Lowenstein-Jensen (LJ) medium.”

*2) Line 105, "one untreated control group of 30 mice and seven treated groups" should be "eight treated groups"!*

Answer: There are indeed a total of 8 groups including 7 treated groups. We changed the text to be clearer.

*3) Line 139-141: For the untreated groups, the entire volume of the footpad suspension was plated onto 10 LJ-media with 0.2 ml each. How did the authors count the CFUs and got 5 to 7 log10 CFU/footpad in some treated groups? As far as I know, it is impossible to count.*

Answer: The reviewer is right, the method for cultivating the treated groups was not clear enough in the manuscript. We modified this part.

*4) What is the definition of their MIC? How did the authors calculate the MICs from the CFU results? Line 160 to 162, this method to test "MIC" was wrong.*

Answer: As classically defined, the Minimum Inhibitory Concentration (MIC) is the lowest concentration of the drug preventing ≥99% of the growth observed on drug free medium. This definition is now included in the text.

*5) “A regimen was considered to be bactericidal if its......”. This was totally wrong!*

Answer: We agree that the term bactericidal may be confusing, particularly when considering the *in vitro* bacteriological definitions. However, this term is commonly used in the Buruli literature (and tuberculosis literature as well) on efficacy of drug regimens in animal model. It refers to a significant decrease in the CFU count in treated animals compared to untreated control ones. We modified the text accordingly.

*6a) When the authors treat the mice in the RIF containing groups, did they separate RIF from other drugs? How long is the duration in between giving RIF and the other drug?*

Answer: Mice treated with regimen containing RIF were first treated with RIF and at least one hour after were treated with other drugs.

*6b) In addition, maybe something wrong as CFUs in the RIF-STR group should be much lower than that from RIF group alone group in table 1. Can these 2 groups mixed?*

Answer: In the first experiment RIF-STR was indeed, as in all our former publications, more active than RIF alone as shown by the CFU counts at 4 weeks (2.15 instead of 4.58, i.e. a 2log difference). Thus, the 2 groups cannot be mixed.

At 8 weeks, cultures were still positive for some mice in both groups. Actually, three additional mice treated by RIF alone were culture positive with a single colony in the entire footpad but were wrongly counted as negative in table 1 since log_10_ of 1 is 0. We corrected the number (5 instead of 2) for the group RIF alone in table 1. Taken as such, data listed in table 1 would indicate no difference between RIF-STR and RIF alone at 8 weeks. However, the remaining CFU numbers were extremely low, 0.47 and 0.57 log_10_ respectively, i.e. corresponding to only ≤5 colonies in the entire footpad. We usually found in the past that both regimens led to culture negativity at 8 weeks. But, as said in the discussion, the inoculum used in the present study was higher, a point that likely explains the residual low number of colonies at 8 weeks.

*7) Many (10) mice died of accident gavage and many (about 30) samples were contaminated, which indicated the performance/skill was not OK. Especially those in the week4 groups in table1, the samples should be diluted and so the contamination should be rare.*

Answer: Regarding the accidents of gavage, they actually represent 2.7% of death among all treated mice, and only ~ 0.1% of treatment days by gavage, rates that are low.

Regarding culture contaminations, they occurred in samples drawn from mice with the most severe lesions (necrosis), almost all from untreated mice of mice treated with the less active drugs. Such samples are heavily contaminated by mice flora (previous personal data) and therefore difficult to be decontaminated without impact on the *M.ulcerans* count by culture. Therefore, in these conditions, contamination of cultures can occur despite using antibiotic-containing culture media. Conversely, contaminations were exceptional in the most active regimens. These comments indicate that contaminations did not result from manipulation errors.

*8) In table 1, in the SEL group, 5 died and 1 contaminated at week4? Is it possible because of the death due to toxicity? At that time point, how about the other 10 mice for the time point week8? If they were still alive, why not kill some of them for the CFU counts at week 4 instead of week 8？Or they died but not indicated?*

Answer: The death of the mice was clearly due to Buruli ulcer infection. Indeed, mice had inflammatory swelling of the whole limb, accompanied by a deteriorating general condition leading to death. We clarified this point in the text.

*9) Why use very low dose of Q230 only at 5 mg/kg?*

Answer: We based our choice on previous studies : (a) Pethe et al, Discovery of Q203, a potent clinical candidate for the treatment of tuberculosis, Nat Med. 2013 Sep;19(9):1157-60): in a murine model of tuberculosis, *M. tuberculosis* CFU was markedly reduced using Q203 at daily doses of 0.4 to 10mg/kg of body weight ; (b) Scherr et all, Targeting the *Mycobacterium ulcerans* cytochrome bc1:aa3 for the treatment of Buruli ulcer, Nat Commun. 2018;9:5370: Q203 was also shown to be active in mouse model against *M.ulcerans* at a dose as low as 0.5 mg/kg daily.

For an administration twice a week, we selected 5mg/kg of body weight, i.e. doses higher than the minimum active doses above. The results obtained demonstrated that Q203-containing regimens were effective at this dose. Maybe lower doses could be effective even administrated twice a week.

*10) Why not test some inactive drugs in vitro first or showing the in vitro data?*

Answer: This comment of the reviewer makes sense at first view. For several of the drugs used, previous published data showed encouraging *in vitro* activity against *M. ulcerans*. For the others, in vitro activity against other mycobacteria such as *M. tuberculosis* suggested potential activity against BU. For these latter drugs we preferred in vivo test for 2 reasons: (a) the determination of the *in vitro* activity against *M.ulcerans* is delicate due to very long time growth (2-3 months); (b) there are past experiences demonstrating discrepancies between *in vitro* and *in vivo* data.

Reviewer #3: See comments below

**Results**

Reviewer #1: *Yes to the questions and the results, figures and tables are easily understandable.*

We thank the reviewer for this positive comment.

Reviewer #2:

*1) The CFU results are not so clear as some mice died or many samples were contaminated.*

Answer: The mean CFU counts were computed from the non-contaminated footpads. In the most groups receiving very active treatment, the contamination numbers were extremely low and therefore would not impair the precision of the results. As underlined above, the contamination occurred mainly in the less active regimen, a fact that could indeed lead to lower precision in CFU counts, but the clinical evolution showed clearly that these regimens are poorly active.

*2) Some results are confusing. For eg, the higher infection doses caused lower MLI. One possible reason is the MLI is too subjective. And CFUs in the STR-RIF group were even more than the RIF alone group. It is very abnormal.*

Answer: Indeed assessing LI is always a little subjective and MLI and mean CFU values are not perfectly proportional. Some antibiotic regimen could even have an impact on inflammation beyond antibacterial activity. It is why CFU counts are the best indicator of *in vivo* antibacterial bactericidal activity.

RIF-STR was, as in all our former publications, more active than RIF alone as shown by the CFU counts at 4 weeks (2.15 instead of 4.58, i.e. a 2 log difference) and by the MLI curves.

At 8 weeks, cultures were still positive for some mice in both groups. Actually, three additional mice treated by RIF alone were culture positive with a single colony in the entire footpad but were wrongly counted as negative in table 1 since the log_10_ of 1 is 0. We corrected the number (5 instead of 2) for the group RIF alone in table 1. Taken as such, data listed in table 1 would indicate no difference between RIF-STR and RIF alone at 8 weeks and, consequently, would suggest discrepancy with MLI. However, the remaining CFU numbers were extremely low, 0.47 and 0.57 log_10_ respectively, i.e. corresponding to only ≤5 colonies in the entire footpad, and thus cannot be compared.

3) In table 2, no any mouse died in the Q203 containing groups but 3 contaminated in RIF-CLR group? For negative CFUs, all undiluted tissue should be plated, and so they can be contaminated even easier. BTW, the relapse rates were evaluated at 20 weeks after treatment stopped. The duration is a little bit shorter.

Answer: Indeed, after 8 weeks of treatment and at the end of the observation period, the entire suspension of the footpads was plated on LJ-media without any dilution. Thus, negative cultures indicate a “real” zero.

The length of post-treatment observation period for relapse assessment varies in the literature between 12 weeks (Converse et al, Plos Negl Trop Dis 2018; Converse et al Antimicrob Agents Chemother 2019), 21 weeks (Converse et al Antimicrob Agents Chemother 2019) and 28 weeks (our previous publication). Actually, analysis of the data of our previous works shows that the relapses after 8 weeks of treatment occurred within the first 20 weeks of post treatment observation period. It is why we decided to take 20 weeks for the current study.

Reviewer #3: See comments below

**Conclusions**

Reviewer #1: *Yes to all questions.*

We thank reviewer one for this positive comment.

*Reviewer #2: The conclusions are OK. However, the findings that the fully intermittent oral regimens containing Q203 can cure BU in 8 weeks are not so valuable and not as good as the fully oral regimens containing Q203 which may cure BU in two weeks. And there are no anythings new except for some other drugs tested showed no activity in vivo.*

Answer: The findings that good results have been obtained by treating BU in mice during 2 weeks are indeed great news. These nice results were based on daily treatments with 3 or 4 drugs. It is difficult to organize DOT on a daily basis in remote places were BU is prevalent. Intermittent DOT is easier to organize and can be conveniently combined with dressing changes, a point that is important from a logistic point of view on the field. In addition, ulcerative wound management by skilled professionals usually takes longer than 2 weeks to reach healing, thus the patients have any way to keep coming to health care centres. Thus, demonstrating good results with 2 administrations per week during 8 weeks is new and medically valuable. BTW, intermittent treatment is, along with shortening the length of treatment, one target in many infectious diseases such as TB.

Finally, it remains to demonstrate that these new treatment administrations (2 weeks daily or 8-week intermittent) are effective in humans. Comparing in the field both strategies would include all aspects of the global management of the patients.

Reviewer #3: See comments below

***Editorial and data presentation***

Reviewer #1: *A minor point is that, although the paper is easy to understand, the English is awkward in places and could do with some editing*

Answer: the manuscript will be corrected by a professional of English editing.

Reviewer #2: (No Response)

Reviewer #3: See comments below

**Summary and General Comments**

Reviewer #1: *These are new and important results from testing combinations of antibiotics that could well be relevant to treatment of Buruli ulcer in humans. There was a surprising improvement in time required to kill all the bacteria, particularly after Q203 containing combinations. The poor results with rifampicin plus clarithromycin (current standard of care treatment in humans) differ from previously published studies but this is discussed by the authors and their explanation is plausible. This does not detract from the value of the paper which represents a valuable contribution to the literature.*

Answer: We thank reviewer one for this positive review.

Reviewer #2: *The authors test several drugs including Q203 targeting QcrB, the component in the ETC. And furthermore, they found that Q203 containing fully intermittent oral regimens can cure M. ulcerans infection effectively. However, the findings are not new to the society of Buruli ulcer treatment field, as cited by this manuscript, (Converse P et al, 2019 doi:10.1128/AAC.00426-19 ), it has been demonstrated that Q203 containing regimen can cure Buruli ulcer in 2 weeks. The total doses are 10 and here at least 18 doses are needed for eight weeks. So the results and conclusion in this paper is not very meaningful. In addition, not only one paper showed the cytochrome bc1-aa3 respiratory terminal oxidase inhibitors were very effective against M. ulcerans infection published in Nature Communications in 2019.*

Answer: The findings that good results have been obtained by treating BU in mice during 2 weeks are indeed great news. These nice results were based on daily treatments with 3 or 4 drugs. It is difficult to organize DOT on a daily basis in remote places were BU is prevalent. Intermittent DOT is easier to organize and can be conveniently combined with dressing changes, a point that is important from a logistic point of view on the field. In addition, ulcerative wound management by skilled professionals usually takes longer than 2 weeks to reach healing, thus the patients have any way to keep coming to health care centres. Thus, demonstrating good results with 2 administrations per week during 8 weeks is new and medically valuable. BTW, intermittent treatment is, along with shortening the length of treatment, one target in many infectious diseases such as TB.

*1) It is better if the authors could provide some pictures of the swelling footpads.*

Answer: We have indeed pictures of swelling footpads (see attached) that could be included.

2) *“0.03 ml of a bacterial suspension containing around 5 log10 Colony Forming Unit (CFU) of the M.ulcerans strain Cu001 (5.02 and 4.6 log10 in the 1st and 2nd experiment, respectively.” “The mean lesion index (MLI) was 2.8 at the start of the treatment in 1 st experiment”. However, MLI was 3 at the start of the treatment in 2nd experiment. According to my experience, the higher infection dose can cause higher MLI. There should be some explanation about why a little bit lower inoculation in the 2nd animal experiment causes a little bit higher the swelling degrees of mice than that in the 1st experiment. In addition, the CFUs at the time of treatment start with higher CFUs in the 2nd experiment.*

Answer: As said above, MLI and CFU values are not perfectly proportional. Actually, the difference between experiences 1 and 2 in term of inoculum (6.93 and 6.87 log10, respectively) and MLI (2.8 and 3, respectively) were very small, and rather not conclusive. As said by reviewer 2, LI is always a little subjective. It is why CFU counts are the best indicator of *in vivo* antibacterial activity.

3) *Line 55 “.” after (WHO) should be deleted.*

Answer: “.” after (WHO) has been deleted.

4) *Line 76 LNZ=LZD?*

Answer: Yes, LNZ=LZD. This mistake has been corrected.

5) *Line 106, abbreviation should be used after it appeared in the context at the first time.*

Answer: This point has been corrected.

6) *Line 204, P< should be italic and there should be a space in between P and <. Similar mistakes in other places.*

Answer: We corrected it at all places.

7) Line 212, *Streptomycine should be “Streptomycin”.*

Answer: We corrected it.

8) *“Buruli ulcer can be successfully treated by a two-month antibiotic combination regimen administered daily” is somewhat right. However, for serious patients, the antibiotic regimens in clinical use are only as adjuvant therapies.*

Answer: Yes indeed. We added in the text “with small-to-moderate size wounds”

Reviewer #3: PNTD 19-01766

*The authors first tested five drug candidates as monotherapy in the mouse footpad model of M. ulcerans infection. The most promising candidate, in terms of bactericidal activity, was Q203. This drug has a long half-life and was tested in a second experiment administered in combination with other long half-life drugs, rifapentine or bedaquiline, in an intermittent, rather than daily, dosing regimen.*

*The results are very interesting and potentially of great interest for the field. However, a number of major and minor concerns are noted and should be addressed. These are enumerated below.*

Answer: We thank reviewer 3 for this extensive and fruitful review and we addressed the concerns enumerated below.

1*. How was the half-life of Q203 determined? Is there a reference for this and also for BDQ and RPT?*

Answer: We added the references in the manuscript, as follows.

- for Q203: Pethe et al. Discovery of Q203, a potent clinical candidate for the treatment of tuberculosis. Nat Med. 2013 Sep;19(9):1157-60;
- for BDQ: Rouan et al. Pharmacokinetics and Pharmacodynamics of TMC207 and Its N-Desmethyl Metabolite in a Murine Model of Tuberculosis. Antimicrob Agents Chemother. 2012;56: 1444–1451. doi:10.1128/AAC.00720-11;
- for RPT: Rosenthal et al. Potent Twice-Weekly Rifapentine-containing Regimens in Murine Tuberculosis. Am J Respir Crit Care Med. 2006;174: 94–101. doi:10.1164/rccm.200602-280OC).

*2. Is the MIC for PBTZ169 against M. ulcerans known? Although the sequence of DprE1 is conserved, it is not 100% identical. There could be conformational differences that would account for the significantly reduced activity. Are the MICs known for the avermectins?*

Answer: Very few works addressed in vitro activity of PBTZ169 against NTMs. *M. marinum*, a species very close to *M. ulcerans*, seems to be susceptible as well as *M. fortuitum* but *M. avium* and *M. abscessus* are resistant. Despite high identity between DprE1 sequence in *M. ulcerans* and *M. tuberculosis* and the conservation of cysteine at the position 38, conformational difference could account for the significantly reduced activity. This point should be addressed by experts in protein conformation.

Avermectin MICs against *M. ulcerans* are as follows:

- selamectin 2-32 mg/l depending on the sources of information;
- ivermectin: 4-64 mg/l depending on the sources of information.

Such ranges concerning *in vitro* data justified our choice to test both compounds *in vivo*. Indeed, in the past, discrepancies have been found between *in vitro* and *in vivo* data.

*3. It is an interesting question as to whether an intermittent regimen facilitates or impedes treatment completion. If dressing changes are less frequent, remembering to take the drugs twice a week could be more problematic.*

Answer: many Buruli ulcer patients with small-to-moderate size wounds are on ambulatory care, and visit healthcare centres twice or three times per week for dressing changes. An intermittent regimen with an administration twice a week of the drugs will allow organizing a directly observed therapy and conveniently combining dressing change and drug administration. We added this point in the discussion.

4. *There are a number of missing (#20-22) and also misaligned references. Please verify the connections throughout the paper. Remove editor name in PNTD refs.*

Answer: References have been entirely revised.

5. *There are disagreements between the text and the tables. For example, the text states that BDQ monotherapy was given 5/7 whereas the table states the more likely 2/7 rhythm.*

Answer: Yes indeed, the BDQ rhythm was 2/7. This point has been corrected.

6. *Which MLI scale was used? Dega et al (14) or Lefrancois et al (15)?*

Answer: The MLI scale used was from Dega et al (ref 14) where the lesion index was defined as follows: index 0: normal footpad; index 1: non inflammatory footpad swelling; index 2: inflammatory footpad swelling; index 3: inflammatory hind foot swelling; index 4: inflammatory leg swelling; and index 5: death of the mouse. We clarified this point in the text.

*7. It is surprising to the reviewers that the swelling values did not go below 1 in either experiment. Please use the same scale for the two figures.*

Answer: Even when the swelling footpad totally regress under treatment, there is still often a little redness indicating scar tissue. As a zero index is defined as a normal footpad, we prefer not to score below “1”. We clarified this point in the text.

*8. It is suggested that for clarity the authors use colour in the graphs since PNTD is an online publication.*

Answer: We used colour for new graphs.

9. *Title: why “fully”? exhibit not exhibited. Thus, “Q203-containing intermittent oral regimens exhibit high sterilizing activity against Mycobacterium ulcerans in mice”.*

Answer: This point has been corrected.

10. *Short title: remove “and” invert Q203 and intermittent. Thus, “Intermittent Q203 oral treatment against Mycobacterium ulcerans in mice”*

Answer: This point has been corrected.

*11. Was a software program used for Mann-Whitney analysis? Please cite. Previous publications by the authors used student T-test. Why did they switch from parametric to non-parametric analysis? Adjustment for multiple comparisons is recommended. Please do chi-square or Fisher’s exact test to compare relapse proportions.*

Answer: There are always discussions about the methods used for comparisons in animal models. It is often advised to use non parametric analysis when there is no normal distribution, e.g. when the number of mice is relatively low.

The software BiostaTGV has been used for Mann-Whitney analysis. As required, we added a Fisher’s exact test to compare relapse proportions that are based on larger numbers of mice. Differences between groups are clearly significant by both analysis methods. This point has been added in “statistical analysis” and “results” sections.

12. *In Table 1, a “.” rather than a “,” should be used for the decimals. Table 2 is correct in this regard. What is the rationale for the Q203 dose selected?*

Answer: This point has been corrected.

We based our choice on previous studies: (a) Pethe et al, Discovery of Q203, a potent clinical candidate for the treatment of tuberculosis, Nat Med. 2013 Sep;19(9):1157-60): in a murine model of tuberculosis, *M. tuberculosis* CFU was markedly reduced using Q203 at daily doses of 0.4 to 10mg/kg of body weight ; (b) Scherr et all, Targeting the *Mycobacterium ulcerans* cytochrome bc1:aa3 for the treatment of Buruli ulcer, Nat Commun. 2018;9:5370: Q203 was also shown to be active in mouse model against *M.ulcerans* at a dose as low as 0.5 mg/kg daily.

For an administration twice a week, we selected 5mg/kg of body weight, i.e. doses higher than the minimum active doses above. The results obtained demonstrated that Q203-containing regimens were effective at this dose. Maybe lower doses could be effective even administrated twice a week.

13. *Consistent use of abbreviations for drugs, not LNZ for LZD. The final e should not be used for selamectin, ivermectin, tedizolid, linezolid, and streptomycin in text and legends.*

Answer: these points have been corrected.

14. *Were suspensions or supernatants of ground suspensions plated (lines 138 and 140)?*

Answer: We plated the suspensions.

15. *In the figure legends, it would be helpful to indicate whether the regimen was 5/7 or 2/7, etc.*

Answer: We added this information in figure legends.

16. *There are grammar and usage errors in the English throughout. For example, when nouns are used as adjectives, the singular form of the noun must be used. Thus, mouse footpads would be correct and should be changed in many places, starting with line 38 in the abstract. Also, mouse model on line 48 and footpad lesions on line 201. Line 300 should be disappointing not deceiving (false friend in French and English). It is highly recommended that a native English speaker correct the manuscript throughout.*

Answer: We corrected these errors as recommended. The manuscript will be corrected by a professional of English editing.

17. *Remove “.” after “(WHO)” (line 55).*

Answer: This point has been corrected.

18. *Tedizolid does not have higher solubility and bioavailability than linezolid. The authors may have misunderstood a statement in the reference cited that referred to improved solubility and bioavailability of the tedizolid phosphate prodrug over tedizolid (lines 75-6).*

Answer: We modified the text as follows: “Tedizolid (TDZ), [7], a new oxazolidinone active against *M. tuberculosis*, sharing the same mechanism of action as linezolid (LZD), a drug active against *M. tuberculosis and M. ulcerans* [8], and exhibiting high bioavailability and long half-life [9]”

19. *“BALB/c”, not “balb/c” (line 88).*

Answer: This point has been corrected.

*20. Where is GVKBio located (line 119)?*

Answer: Q203 has been synthetized by GVKBio (GVK Biosciences Private Limited, Hyderabad, India) and kindly provided by K. Pethe. The text has been corrected accordingly.

21. *Were the lesion indexes measured weekly (line 143)?*

Answer: Yes, the lesion indexes were measured weekly. We added this point it in the text.

22. *“Oleic acid-Albumin-Dextrose-Catalase” (line 157).*

Answer: This point has been corrected.

23. *Please confirm use of dimethylformamide, not dimethylsulfoxide?*

Answer: We used dimethylformamide.

24. *In Table 1, there is no footnote “c” between “b” and “d”.*

Answer: This point has been corrected.

25. *How was it determined that mice died from M. ulcerans infection (lines 220-1)?*

Answer: The text is indeed not clear enough and was modified as follows: ”Mice with extensive BU lesions (inflammatory swelling of the whole limb, accompanied by a deteriorating general condition) either died or had to be sacrificed.”

26. *In Table 2, it states that the swelling index was between 2 and 3. However, line 224 states and the figure shows 3. Please correct.*

Answer: This point has been corrected in Table 2.

27. *Table 2 is also misleading in results at 28w were not “during treatment” as suggested by the table heading.*

Answer: Yes indeed. We modified Table 2 by adding “Results after observation period”.

28. *In Table 2, the CFU count for Q203 2/7 at W2 has an extra “0.”*

Answer: This point has been corrected.

29. *Line 248: “where”, not “were”*

Answer: This point has been corrected.

30. *Please provide references for the available PK data (lines 290-291).*

Answer: We verified the references that appear in the text.

31. *Line 298: “tedizolid” or “TDZ”, not both*

Answer: This point has been corrected.

32. Line 314: delete “Although”

Answer: This point has been corrected.

*33. Line 334: Is implantation rather than inoculum meant here? In addition, was footpad swelling also greater than usual before the onset of treatment in this experiment?*

Answer: The text is indeed not clear enough. Actually, the CFU counts at the time of treatment start were higher than in our previous studies. The text has been corrected and “inoculum” has been replaced by “bacterial load”.

34. *Line 350: probably effective rather than efficient.*

Answer: This point has been corrected.

35. *Did the authors see any evidence of carryover of Q203 or other long half-life drugs? It may useful to readers to include a statement about the potential for drug carryover and whether it appeared to be mitigated by use of LJ.*

Answer:

- for the CFU counts at 4 and 8 weeks of treatment, mice were let 2-5 days without treatment before sacrifice to decrease the risk of carry over.
- for the CFU counts after post treatment observation period, the length of time without treatment was 20 weeks, making rather improbable any carryover.

In addition, footpad biopsies (weighing ~0.02 g.) were homogenised in 2 ml Hank solution (i.e. 1/100 dilution) and plated on LJ, a medium that is known to mitigate carry over effect (see Lounis et. al. J. Clin. Microbiol. 2008, 46, 2212-2215). Taken together, these 2 additional facts most likely rule out the possibility of carryover.
