# Supplementary material for: Telacebec (Q203)-containing intermittent oral regimens sterilized mice infected with Mycobacterium ulcerans after only 16 doses
Source: PLoS Negl Trop Dis. 2020 Aug 31;14(8):e0007857. doi: 10.1371/journal.pntd.0007857 (PMC7494103; doi:10.1371/journal.pntd.0007857)
Supplement: Attachment — Submitted filename: Response to reviewers_PNTD D-19-01766.docx [file pntd.0007857.s002.docx]

**Responses to reviewer’s comments**

**Methods**

**Are the objectives of the study clearly articulated with a clear testable hypothesis stated?</br> -Is the study design appropriate to address the stated objectives?</br> -Is the population clearly described and appropriate for the hypothesis being tested?</br> -Is the sample size sufficient to ensure adequate power to address the hypothesis being tested?</br> -Were correct statistical analysis used to support conclusions?</br> -Are there concerns about ethical or regulatory requirements being met?**

**Reviewer #1**: Please see previous report

**Reviewer #3**: The methods are now acceptable for this reviewer.

Answer: We thank the reviewer for this positive comment.

**Reviewer #4**: This is a well-designed, carefully performed animal experiment allowing for straightforward conclusions to be drawn; the number of experimental animals have been accounted for, and is likely to be justified to be able to reach at the conclusions; the paper adds to what we know about Q203 (why not use the new name telacebec?) and many questions regarding the potential benefit of intermittent therapy have been addressed, carrying out many control experiments in the same model. All failed experiments have been accounted for, making the paper very convincingly robust.

Answer: We thank the reviewer for this positive comment. We changed Q203 by telacebec (Q203)

**Reviewer #5**: The objectives of the study are clearly articulated with a clear testable hypothesis stated. Also, the study design was appropriate to address the stated objectives.

The population was clearly described and appropriate for the hypothesis being tested, and the sample size was sufficient to ensure adequate power to address the hypothesis being tested. Correct statistical analysis were used to support conclusions but specific p values were not presented. Concerns about ethical or regulatory requirements were met. Below are some specific statements.

Answer: Specific p values have been presented

1. Rewrite lines 103 – 104 to read: The mice were randomly allocated into eight groups (1st experiment) and ten groups (2nd experiment) using a randomization table generated by the web site Randomization.com (<https://www.randomization.com>).

Answer: We modified the text as recommended.

2. Lines 106 and 110. Write “1st experiment” as “First experiment” and 2nd Experiment as “Second experiment” since they are beginning the sentence.

Answer: We modified the text as recommended.

3. Line 105: Please what do you mean by: The groups were as follows (drug, dosage, number of doses/week)???

Answer: We modified the text as follows to be more comprehensible: “The groups were treated (with respect to drug, dosage, as well as number of doses/number of weeks, respectively) as follows”

4. Lines 178 – 179: Please provide a reference for your MIC definition.

Answer: We added the reference in the manuscript: Inderlied CB, Nash KA. Antimicrobial agents: in vitro susceptibility testing, spectra of activity, mechanisms of action and resistance, and assays for activity in biologic fluids. 4th ed. Antibiotics in laboratory medicine. 4th ed. The Williams & Wilkins Co., Baltimore. Md: M. D. V. Lorian; 1996. pp. 127–175.

5. Line 197: Heading should be “First experiment” instead of 1st experiment

Answer: We modified the text as recommended.

6. Why are you not providing the specific p values? You mostly quoted P values as (p <0.05) line 217 and 271, (p < 0.001) line 225, (p ≤0.01), line 267 and (p <0.002) line 282. Please try and provide the specific P values, E.g (p = 0.012).

Answer: Specific p values have been presented

**RESULTS**

**Does the analysis presented match the analysis plan?</br> -Are the results clearly and completely presented?</br> -Are the figures (Tables, Images) of sufficient quality for clarity?**

**Reviewer #1:** Yes

**Reviewer #3:**

Fig. 1: The reviewer finds (A) lesion index 1 not typical of grade 1 swelling; more like, 0-1, improving after treatment. Other images for grades 2, 3, and 4 are fine.

Answer: We added to the figure a 1^st^ image to show a lesion index 0.

Line 139: why not 0-1?

Answer: There is still often a little redness indicating scar tissue and no return to the baseline. As the zero index is defined as a fully normal footpad, we preferred to score this stage as “1”.

Table 1: Please indicate 2.8± SD at start of treatment

Answer: We have added SD

Line 245: 3±SD

Answer: We have added SD

Line 265: the CFUs were not unchanged but you could say stable

Answer: Unchanged has been replaced by table

**Reviewer #4:** results are well presented, and graphics and tables are clear.

**Reviewer #5:**

1. The analysis presented matched the analysis plan. The results were clearly and completely presented but the figures (Images) are not of sufficient quality for clarity. Quality of the figures need to be improved.

Answer: We improved the figure and they passed the test with PACE.

2. Figure 1: Authors need to calculate and show the image sizes to help with comparison of the Lesion index grades

Answer: The scale has been added on the pictures

3. Figures 2 and 3: On the Y-axis, authors have use comma (,) instead of point (.). Please correct throughout. E.g. 1,0, 1,5, and 2,0, should be 1.0, 1.5 and 2.0.

Answer: We corrected.

**CONCLUSIONS**

**Are the conclusions supported by the data presented?</br> -Are the limitations of analysis clearly described?</br> -Do the authors discuss how these data can be helpful to advance our understanding of the topic under study?</br> -Is public health relevance addressed?**

**Reviewer #1:** Yes

**Reviewer #3:**

Line 325: Are mice good animal models for avermectins? These drugs are very potent at low doses in humans for parasites and scabies mites but much higher doses are needed in mice and may be toxic.

Answer: Indeed, doses used in animals and humans are different.

For the purpose of evaluating avemectin activity against *M.ulcerans*, we aimed to obtain AUC in mice as close as to the AUC in human. Based on different works (references underneath) on ivermectin and selamectin we concluded that dose should be 12mg/kg in mice for obtaining AUC in mice as close as that in human.

(Scherr N, Pluschke G, Thompson CJ, Ramón-García S. Selamectin Is the Avermectin with the Best Potential for Buruli Ulcer Treatment. Johnson C, editor. PLoS Negl Trop Dis. 2015;9: e0003996. doi:10.1371/journal.pntd.0003996; Geyer J, Gavrilova O, Petzinger E. Brain penetration of ivermectin and selamectin in mdr1a,b P-glycoprotein- and bcrp - deficient knockout mice. J Vet Pharmacol Ther. 2009;32: 87–96. doi:10.1111/j.1365-2885.2008.01007.x; Omansen TF, Porter JL, Johnson PDR, van der Werf TS, Stienstra Y, Stinear TP. In-vitro Activity of Avermectins against Mycobacterium ulcerans. Johnson C, editor. PLoS Negl Trop Dis. 2015;9: e0003549. doi:10.1371/journal.pntd.0003549)

In addition, according to a study of Bishop et al. selamectin is well tolerated in mice up to 300mg/kg. (Bishop BF, Bruce CI, Evans NA, Goudie AC, Gration KAF, Gibson SP, et al. Selamectin: a novel broad-spectrum endectocide for dogs and cats. Vet Parasitol. 2000;91: 163–176. doi:10.1016/S0304-4017(00)00289-2).

Line 358: The authors might note that relapse occurred at similar proportions in other mouse studies:

Almeida et al. after 4 weeks treatment in a kinetic rather than curative model, PMID: 21245920 Converse et al. after 8 weeks treatment in a curative model, Figs 3& 4, PMID: 26042792 Converse et al. after 6 weeks treatment in a curative model, Fig. 2, PMID: 30102705

Answer: We included the reference Converse Plos Negl Trop Dis 2015 and added a sentence on the relapse rate found by these authors 20 weeks after treatment completion by RIF-CLR 5/7.

**Reviewer #4:** conclusions are all supported by the data presented; limitations (especially, using high inoculums resulting in persisting live bacteria after 8 wk treatment) have been addressed; the fact that only one wild-type M ulcerans isolate was used was not mentioned as a potential weakness, but this does not seem a very important limitation anyway; the authors have a clear mindset to help advance treatment of Buruli ulcer in rural Africa; and they help the reader how to understand some of the scientific questions regarding drug treatment of M ulcerans infection.

Answer: We thank the reviewer for these positive comments.

**Reviewer #5:**

1. Line 336: The authors indicated that RIF and RIF-STR were highly bactericidal as in all their preceding works [20]. Meanwhile they cited just one publication (Ref 20).

Answer: We added a second reference to the text (Ji B, Chauffour A, Robert J, Jarlier V. Bactericidal and sterilizing activities of several orally administered combined regimens against Mycobacterium ulcerans in mice. Antimicrob Agents Chemother. 2008;52: 1912–1916. doi:10.1128/AAC.00193-08)

2. The authors tested several drugs including Q203 (now named telacebec) and thus observed that Q203-containing intermittent oral regimens can cure M. ulcerans infection effectively. However, the findings are not novel with regards to Buruli ulcer treatment. A publication by Converse et al, 2019 (doi:10.1128/AAC.00426-19) observed that footpad swelling decreased more rapidly in mice treated with Q203-containing regimens than in mice treated with RIF and STR (RIF+STR) and RPT and CFZ (RPT+CFZ). In that study nearly all footpads were culture negative after only 2 weeks of treatment with regimens containing RPT, CFZ, and Q203. No relapse was detected after only 2 weeks of treatment in mice treated with any of the Q203-containing regimens. In contrast, 15% of mice receiving RIF+STR for 4 weeks relapsed. Converse et al (2019) concluded that it may be possible to cure patients with Buruli ulcer in 14 days or less using Q203-containing regimens rather than currently recommended 56-day regimens.

In the current study under review, as mentioned earlier, the authors evaluated the bactericidal activity of several new antimicrobials drugs in a mouse model of BU and found that the Q203 exhibited the highest bactericidal effect. They also subsequently identified new antibiotic combinations containing Q203 with high sterilizing activity when administrated twice a week for 8 weeks, i.e. for a total of only 16 doses. The authors need to highlight the latter more and indicate the new combinations to bring out the novelty of this study rather than portraying the already established idea that Q203-containing intermittent oral regimens exhibit high sterilizing activity against Mycobacterium ulcerans in mice. Thus, I recommend a modification to the title and key findings should also be based more on the new Q203-containing combinations identified.

Answer: We accordingly modified the title and key findings at the beginning of the discussion section

REF:

Converse, P. J., Almeida, D. V., Tyagi, S., Xu, J., & Nuermberger, E. L. (2019). Shortening Buruli Ulcer Treatment with Combination Therapy Targeting the Respiratory Chain and Exploiting Mycobacterium ulcerans Gene Decay. Antimicrobial agents and chemotherapy, 63(7), e00426-19.

3. The study by Converse et al, 2019 is very instrumental with regards to the current study under review; meanwhile, the authors cited that study just once (at the last few sentences of the discussion). There are a number of areas in the earlier sections that this reference needed to be mentioned.

Answer: The study by Converse has been introduced in the introduction section and earlier in the discussion section, i.e. in a total of 3 places.

4. Lines 371 -381: Authors need to give a more detailed contrast of the current study and that of Converse et al, 2019. What has been indicated there is not enough.

Answer: The potential interest of the intermittent 2 drugs regimens and the contrast with the daily 3-4 drugs regimen experimented by Converse has been detailed at the end of the discussion section.

5. Lines 379 -380: “thanks to the long half-life and good bio availabilities of these drugs” needs to be re-written as it is not a proper way of scientific writing.

Answer: We modified the text as follows: “due to the long half-life and good bioavailability of these drugs”

6. Generally, the conclusions based on the major findings are OK, however, I am not so sure of the novelty of major findings highlighted by the authors.

Answer: The major findings and the novelty of the present study that the double combinations containing Q203 and bedaquiline or rifapentine is highly bactericidal and sterilizing when administrated twice a week for 8 weeks, i.e. for a total of only 16 doses.

Indeed, Q203 is already known to be active against BU in mice, through our previous study and that by Converse. However, an intermittent scheme of treatment would greatly simplify the treatments of BU in many places in the field where patients living in remote areas visit healthcare centers on an ambulatory care basis (twice or three times per week) for dressing changes, a rhythm that could allow receiving supervised intermittent antibiotic administration. Searching for efficient intermittent treatments is also a challenge for the two other major mycobacterial infections, tuberculosis and leprosy.

**EDITORIAL AND DATA PRESENTATION MODIFICATIONS**

**Reviewer #1**: Minor revision of the use of English

Answer: The manuscript was corrected by a professional of English editing.

**Reviewer #3:**

**Abstract**

Line 31: delete “the”

Answer: We modified the text as recommended.

Line 34: (an oxazolidinone …) … (avermectin compounds)

Answer: We modified the text as recommended.

Author summary Line 51: the verb form should be administered. Please change throughout manuscript. Or, administering, as appropriate.

Answer: We modified the text as recommended.

**Introduction**

Line 61: patient adherence

Answer: We modified the text as recommended.

Lines 82 and 85: Please cite Pethe et al. then Rosenthal et al and Rouan et al here, too.

We introduced the references on long half-lives of Q203 line 82 as well as of BDQ and rifapentine line 85

Answer: We modified the text as recommended.

**Methods**

Line 88: 4 week-old

Answer: We modified the text as recommended.

Line 119: Is this the correct affiliation for Dr. Cole?

Answer: We completed his affiliation according to his last manuscript referenced in PubMed.

Line 129: in the same volume

Answer: We modified the text as recommended.

Line 142: ground in …… in a final

Answer: We modified the text as recommended.

Line 160: 8 week

Answer: We modified the text as recommended.

Line 162 and 163: for instead of during

Answer: We modified the text as recommended.

Line 171: McFarland

Answer: We modified the text as recommended.

Line 187 and 188: Ethics/ethics. The experiment project

Answer: We modified the text as recommended.

**Results**

Line 208: Line 258: Fig 2. First experiment: Change in mean lesion index of M. ulcerans-infected mouse footpads during treatment for 8 weeks

Answer: We modified the text as recommended.

Line 217: mouse group

Answer: We modified the text as recommended.

Line 222: lesions Table 1 b,e,i: advanced lesions ; c and d: due a gavage accident

Answer: We modified the text as recommended.

line 246: swelled

Answer: We modified the text as recommended.

**Fig 3**

Line 258: Fig 3. Second experiment: Change in mean lesion index of M. ulcerans-infected mouse footpads during and after treatment for 8 weeks

Answer: We modified the text as recommended.

Line 260: Dosages were as follows:

Answer: We modified the text as recommended.

Line 267: the untreated group.

Answer: We modified the text as recommended.

**Table 2**

a: treatment began

c,e: advanced lesions

e,f: due a gavage accident

Answer: We modified the text as recommended.

**Discussion**

Line 322: The same

Answer: We modified the text as recommended.

Line 326: humans

Answer: We modified the text as recommended.

Line 349: administering twice weekly for 8 weeks (similar correction line 352-353)

Answer: We modified the text as recommended.

Line 356: with low CFU counts

Answer: We modified the text as recommended.

Line 368: The reason for this

Answer: We modified the text as recommended.

Line 371: italicize bc and aa

Answer: We modified the text as recommended.

Line 380: bioavailability I suggest that the first line of the Discussion section should be changed to reflect the preponderance of current expert opinion on Buruli ulcer treatment. Therefore, it should be changed accordingly:

“Although nearly all Buruli ulcer lesions can be successfully treated by a two-month antibiotic combination regimen administered daily,”

Answer: We modified the text as recommended

The authors may wish to cite Wadagni et al. PMID: 31658295 and/or PMID: 29605498 as well as Clinicaltrials.gov: NCT01659437. I believe the latter study may (soon) be in press in the Lancet.

Answer: Reference Wadagni has been introduced

**Reviewer #4:** This is good science, presented in a clear fashion; and the only thing I see as something that might be improved is the use of prepositions and the English language article 'the' but it does not interfere with the clarity of the text.

Answer: We thank the reviewer for the positive comment. The manuscript was corrected by a professional of English editing.

**Reviewer #5:**

1. The authors indicated that “Buruli ulcer (BU), caused by Mycobacterium ulcerans, was only treated by surgery until 2004”. Is this on a worldwide scale? If Yes, please indicate. Also, this statement sounds too emphatic and thus would require reference(s) to support, as done for the statement which showed the first medical treatment of Buruli ulcer recommended by the World Health Organization.

Answer: We added the WHO reference “Treatment of Mycobacterium ulcerans disease (Buruli ulcer): guidance for health workers” https://apps.who.int/iris/bitstream/handle/10665/77771/9789241503402_eng.pdf;jsessionid=AECF9887A2910FB44C46C2212817A90F?sequence=1

2. Lines 64 – 67: Please provide references for the stamen below:

Answer: For instance, many Buruli ulcer patients with small-to-moderate size wounds are on ambulatory care [REF], and visit healthcare centers twice or three times per week for dressing changes [REF], a rhythm that could allow receiving supervised intermittent antibiotic administration.

Answer: We added a reference seeing that most patients in remote areas are treated on an ambulatory basis (Phillips RO, Robert J, Abass KM, Thompson W, Sarfo FS, Wilson T, et al. Rifampicin and clarithromycin (extended release) versus rifampicin and streptomycin for limited Buruli ulcer lesions: a randomised, open-label, non-inferiority phase 3 trial. Lancet Lond Engl. 2020;395: 1259–1267. doi:10.1016/S0140-6736(20)30047-7)

3. The entire manuscript needs English Grammar editing and proofreading. E.g.

Answer: The manuscript was corrected by a professional of English editing.

Line 88. There should be a comma (,) after the word “Respectively”. Also, line 88 -89, since 1st and 2nd were appearing for the first time they need to be written in full as First (1st ) and second (2nd) experiments.

Answer: We modified the text as recommended.

**SUMMARY AND GENERAL COMMENTS**

**Use this section to provide overall comments, discuss strengths/weaknesses of the study, novelty, significance, general execution and scholarship. You may also include additional comments for the author, including concerns about dual publication, research ethics, or publication ethics. If requesting major revision, please articulate the new experiments that are needed.**

**Reviewer #1:** See below

**Reviewer #3:** The manuscript is much improved but there are editorial issues remaining that should have been addressed in the first revision.

Line 306: I am not sure that successful treatment with antibiotics is restricted to small-to-moderate lesions. Even edematous lesions have been reported to be cured in the various clinical trials.

Answer: We changed the 1^st^ sentence of the discussion section as required.

**Reviewer #4**: I personally like a Discussion section starting with the most striking finding from the work presented ('this study provides evidence of excellent healing of M ulcerans infection without relapse in the mouse model, using the novel agent telacebec in a twice-weekly regimen for only 8 weeks'. But, as this is the report on an animal experiment, perhaps the current Discussion is good enough for readers that are used to read papers on experimental research. PLoS NTD has however also a clinical readership; no doubt, clinicians pick up the important message from the title as well.

Answer: We accordingly modified the title and key findings at the beginning of the discussion section

**Reviewer #5**: The current study used a well-established mouse model of M. ulcerans infection which can be considered a useful guide to treatment of human infection. Ethical issues have been adequately addressed. However, there are several major issues which need to be addressed before this manuscript can be considered for publication. Also, I am not so sure of the novelty of major findings highlighted by the authors and significance of the study was not highlighted very much in the writing.

The major findings and the novelty of the present study that the double combinations containing

Q203 and bedaquiline or rifapentine is highly bactericidal and sterilizing when administrated twice a week for 8 weeks, i.e. for a total of only 16 doses.

Answer: Indeed, Q203 is already known to be active against BU in mice, through our previous study and that by Converse. However, an intermittent scheme of treatment would greatly simplify the treatments of BU in many places in the field where patients living in remote areas visit healthcare centers on an ambulatory care basis (twice or three times per week) for dressing changes, a rhythm that could allow receiving supervised intermittent antibiotic administration. Searching for efficient intermittent treatments is also a challenge for the two other major mycobacterial infections, tuberculosis and leprosy.
